# The Effects of Smoking on Human Pharynx Microbiota Composition and Stability

**DOI:** 10.1128/spectrum.02166-21

**Published:** 2023-02-14

**Authors:** Lydia Bach, Asha Ram, Umer Z. Ijaz, Thomas J. Evans, Daniel T. Haydon, Jan Lindström

**Affiliations:** a School of Biodiversity, One Health and Veterinary Medicine, University of Glasgow, United Kingdom; b School of Science and Engineering, University of Glasgow, United Kingdom; c School of Infection and Immunity, Glasgow Biomedical Research Centre, University of Glasgow, United Kingdom; University of Michigan-Ann Arbor

**Keywords:** microbiota, smoking, community stability, perturbation, invasion, cold, antibiotics, microbiome, oropharynx, stability

## Abstract

The oral microbiota is essential to the health of the host, yet little is known about how it responds to disturbances. We examined the oropharyngeal microbiota of 30 individuals over 40 weeks. As the oropharynx is an important gateway to pathogens, and as smoking is associated with increased incidence and severity of respiratory infections, we compared the microbiota of smokers and nonsmokers to shed light on its potential for facilitating infections. We hypothesized that decreased species diversity, decreased community stability, or increased differences in community structure could facilitate invading pathogens. We found that smoking is associated with reduced alpha diversity, greater differences in community structure, and increased environmental filtering. The effects of short-term perturbations (antibiotic use and participants exhibiting cold symptoms) were also investigated. Antibiotic use had a negative effect on alpha diversity, irrespective of smoking status, and both antibiotic use and cold symptoms were associated with highly unique bacterial communities. A stability analysis of models built from the data indicated that there were no differences in local or global stability in the microbial communities of smokers, compared to nonsmokers, and that their microbiota are equally resistant to species invasions. Results from these models suggest that smoker microbiota are perturbed but characterized by alternative stable states that are as stable and invasion-resistant as are the microbiota of nonsmokers. Smoking is unlikely to increase the risk of infectious disease through the altered composition and ecological function of the microbiota; this is more likely due to the effects of smoking on the local and systemic immune system.

**IMPORTANCE** Smoking is associated with an increased risk of respiratory infections. Hypothetically, the altered community diversity of smokers’ pharyngeal microbiota, together with changes in their ecological stability properties, could facilitate their invasion by pathogens. To address this question, we analyzed longitudinal microbiota data of baseline healthy individuals who were either smokers or nonsmokers. While the results indicate reduced biodiversity and increased species turnover in the smokers’ pharyngeal microbiota, their ecological stability properties were not different from those of the microbiota of nonsmokers, implying, in ecological terms, that the smokers’ microbial communities are not less resistant to invasions. Therefore, the study suggests that the increased propensity of respiratory infections that is seen in smokers is more likely associated with changes in the local and systemic immune system than with ecological changes in the microbial communities.

## INTRODUCTION

The oral microbiota comprises a large number of species that are found in high densities, occurring in several distinct habitats, including the teeth, tongue, buccal mucosa, and oropharynx (1). It is well-established that a stable microbiota contributes to a healthy status for the host (2). Correspondingly, changes in community composition and abundance within these microbial communities are associated with the pathogenesis of several diseases (3). These changes can be further linked with environmental stimuli that affect colonization and growth, specifically, press perturbations that stay in place for a long time and pulse perturbations that tend to be of short duration (4).

Smoking is an example of a press perturbation that has been shown to change the oral microbial community and promote pathogenetic species (5–8). Indeed, a major health outcome of smoking is the increased propensity for infections (9). There are three possible pathways of how smoking-related changes in the oropharyngeal microbiota could be associated with infection risk: changes in microbiota merely reflect a changed immune response in smokers, microbiota play an active role in deferring or facilitating invasions of pathogens, or both of these processes are involved. It is well-established that the harmful components in smoke mediate the host immune response, affecting, in particular, humoral and cell-mediated responses, which consequently affects the microbial community composition (10–13). Moreover, smoking has also been shown to affect many cellular processes, including neutrophil chemotaxis, adherence, phagocytosis, and function (14–17). It has been hypothesized that the main cause of the increased infection risk is associated with a compromised anti-bacterial function of leukocytes, including neutrophils, monocytes, T cells, and B cells (18), as well as pathogen enrichment (8, 19). In addition, the toxic substances contained in cigarette smoke, as well as oxygen deprivation, have been hypothesized to contribute to the altered microbiota in smokers (6, 20). Observational and interventional studies have found that the composition of the intestinal microbiota is also altered in smokers, with some taxa occurring in higher abundances (e.g., Parvimonas, Fusobacterium, Campylobacter, Bacteroides, and Treponema), while others have been shown to decrease in abundance (e.g., Veillonella, Neisseria, and Streptococcus) (7, 8, 20). However, there are also indications that the increased infection risk in smokers could be more directly linked to the changes in the oropharyngeal microbiota, as the changed community might be more prone to invasions by pathogens (9, 21, 22). It remains to be determined how smoking affects the stability and invasibility of these communities in ecological terms.

In addition to the potential effects of long-term perturbations, such as smoking, short-term perturbations can affect the oral microbiota. Examples of pulse perturbations in microbiota include short-term antibiotic use or the common cold. Much of the work investigating the impact of antibiotics on microbial communities has been conducted in the gut, with findings relating to the oral microbiota remaining ambiguous (23, 24). Those focusing on the oral microbiota showed that antibiotics can cause shifts in the microbial community composition and reduce microbial diversity (25, 26). These effects are short-lived, which the authors suggested may be associated with a greater resilience of oral, compared to gut, communities (24, 27).

Viral infections, such as the common cold, are gaining interest in the microbiota literature (28–30), but few studies have investigated their impact on the human oropharyngeal microbiota (31). Evidence suggests that infection with Rhinovirus, one of the viruses that is responsible for the common cold, also results in increases in Neisseria and Haemophilus in the nasopharynx, which possibly leads to secondary bacterial infections (32).

While evidence supports the individual impacts of these perturbations on the oral microbiota, the purpose of the present investigation is to examine the combined effects of smoking, antibiotic use, and the acquisition of a common cold on the microbial community in terms of community composition, alpha diversity, beta diversity, and environmental filtering (i.e., abiotic factors preventing species from persisting in a location) (33). Going beyond those traditional measures, we also investigated stability (local and global stability) and invasibility in smokers and nonsmokers, providing us with a deeper understanding of the microbial community dynamics that are associated with pulse and press perturbations.

The relationship between diversity and ecological stability has been a long-standing question in ecology (34–36). Some studies suggest that more diverse and complex communities tend to be more stable and are thus generally more resistant to perturbations (37). Species removals or additions (e.g., through invasions), can result in major shifts in community structure and dynamics, which can thereby result in changes in community stability and function. In the context of the microbiota, this stability is critical for the well-being of the host, as it ensures that beneficial taxa persist over long periods, providing vital ecosystem functions (38–41).

Here, we test four specific perturbation-related hypotheses. We hypothesize that (i) smoking is equivalent to an ecological perturbation that is likely to decrease alpha diversity and cause greater differences in community structure and filtering; (ii) the community composition of the smoker microbiota will be different from that of nonsmokers; (iii) thereby, microbial communities in smokers will be less stable (locally and/or globally); (iv) meaning that the microbial communities in smokers will be more invasible, compared to those of nonsmokers.

## RESULTS

The microbial communities were comprised of 9 phyla, 202 genera, and 1,438 assignments at the operational taxonomic unit (OTU) level. At the phylum level, 99.7% of reads were taxonomically classified, with the remaining 0.3% belonging to unknown or unclassified bacteria. Firmicutes, Bacteroidetes, and Proteobacteria dominated both the smoker and nonsmoker communities.

**FIG 1:**
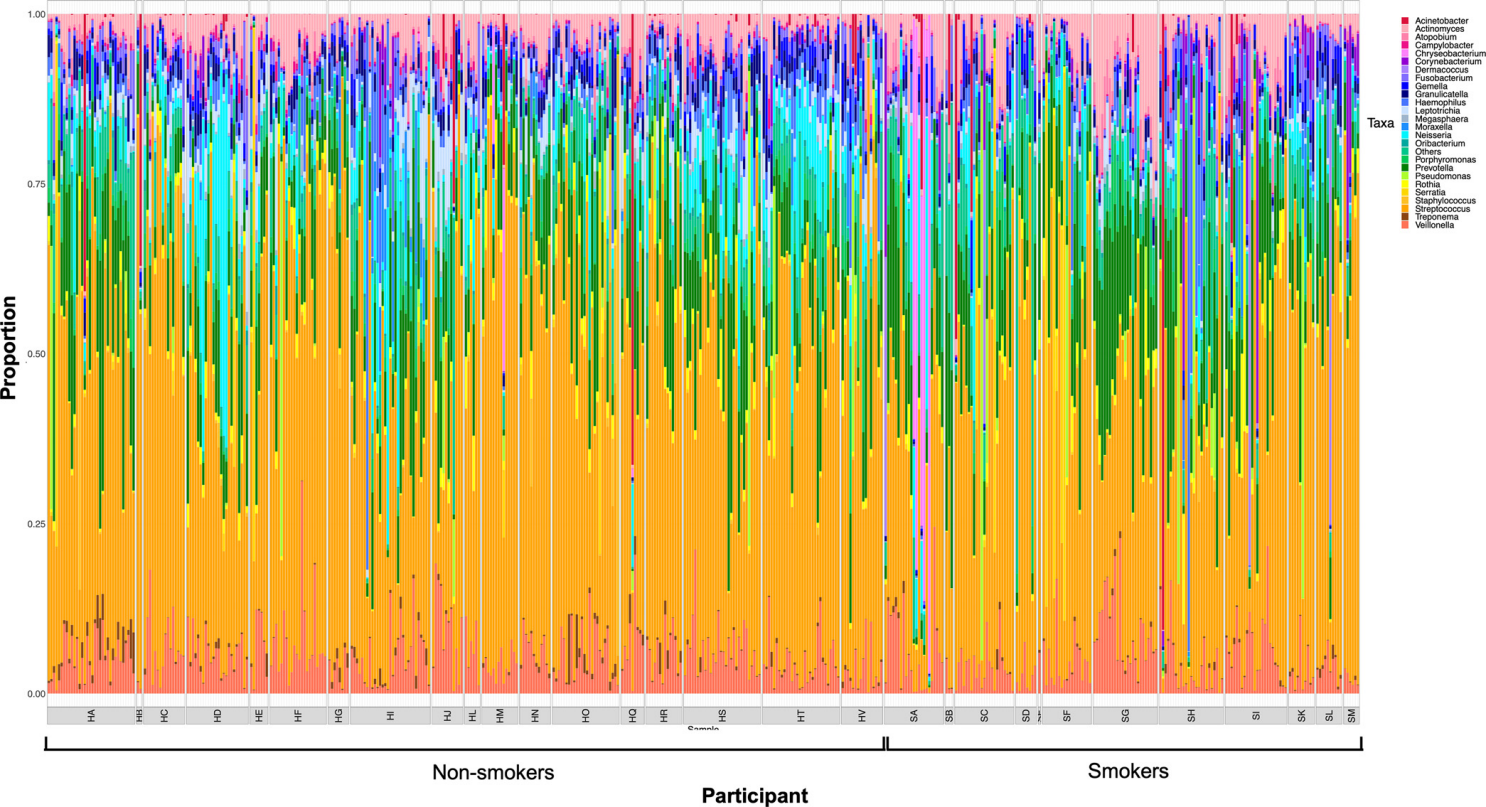
Relative abundance (%) of the 25 dominant genera for smokers and nonsmokers for each week over the sampling period.

The relative abundances of the 25 most abundant genera were similar between time points for each participant, and they were broadly comparable across participants, irrespective of smoking status (Fig. 1). The three most dominant genera in healthy smokers and nonsmokers were Streptococcus (mean: 0.470, SD: 0.011), *Prevotella* (mean: 0.094, SD: 0.004), and *Veillonella* (mean: 0.058, SD: 0.002).

The most abundant OTUs in healthy smokers belonged to Streptococcus species, reflecting the general abundance of Firmicutes. These included Streptococcus mitis, Streptococcus salivarius, and Streptococcus parasanguinis, which were also the most dominant OTUs in the healthy samples from nonsmoking participants. Further details on the microbial composition of nonsmokers can be found in our previous publication (42). For plots showing the temporal changes in the community composition for all participants over the course of the study period at the phylum, family, and OTU levels, refer to Fig. S2, Fig. S3, and Fig. S4, respectively.

**FIG 2:**
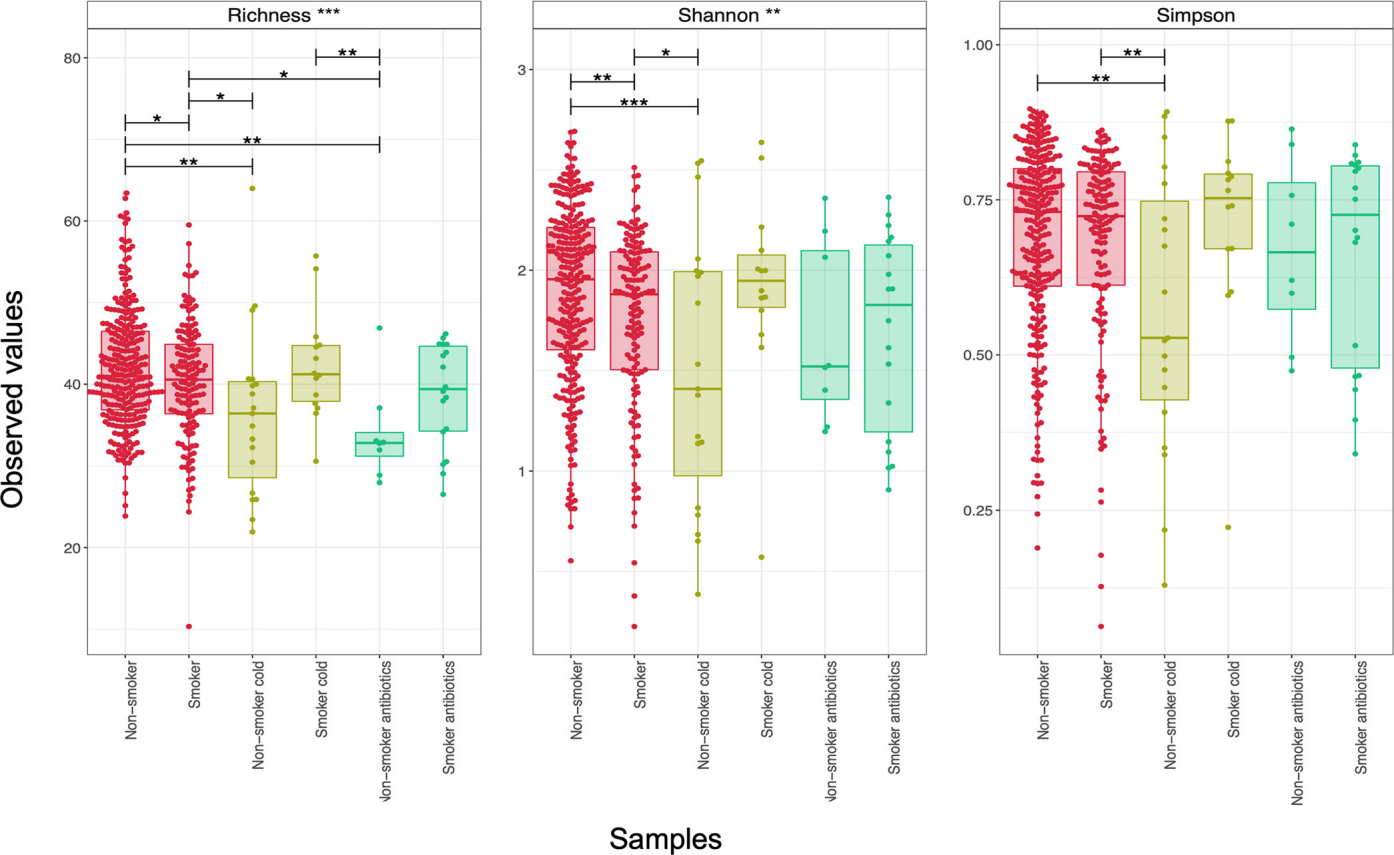
Three alpha diversity measures: species (OTU) richness, Shannon diversity, and Simpson diversity, calculated based on the oropharyngeal microbiota of smokers and nonsmokers when healthy, expressing cold symptoms, or taking antibiotics. The lines connect two categories if the values are significant per an ANOVA, with one within subject factor accounting for the participant IDs.

Alpha diversity was significantly higher in healthy nonsmokers, compared to healthy smokers (Fig. 2). Alpha diversity was significantly lower in nonsmokers exhibiting cold symptoms, compared to healthy nonsmokers. However, there were no differences in alpha diversity between healthy smokers and smokers experiencing cold symptoms, suggesting that cold had little or no effect on alpha diversity in smokers. Antibiotic use resulted in a decreased diversity in nonsmokers and smokers (with the exception of Simpson’s index), but the use of antibiotics resulted in a greater similarity in alpha diversity measures between smokers and nonsmokers.

A beta dispersion analysis suggested that data homogeneity differed significantly with all factors (smoking status: smoker/nonsmoker, sex, age, and participant), with individual participants of the study being the single most important factor explaining the data variability for Bray Curtis (*R*^2^: 0.131%), unifrac (*R*^2^: 0.159), and weighted unifrac (*R*^2^: 0.132%,), and this was followed by health status (*R*^2^ between 0.054 and 0.065 for Bray-Curtis, unifrac, and weighted unifrac), whereas all of the other factors contributed significantly less (Table 1).

**TABLE 1:** Statistics for beta dispersion comparison, based on Bray-Curtis, unifrac, and weighted unifrac, based on a PERMANOVA analysis

Measure	Factor	Degrees of freedom	Sum of squares	Mean square	F	*R* ^2^	Pr(>F)
Bray-Curtis	Health status	5	7.98	1.60	6.09	0.054	0.001
	Sex	1	0.82	0.82	3.11	0.006	0.002
	Age	1	1.57	1.57	5.96	0.011	0.001
	Participant	28	19.32	0.69	2.63	0.131	0.001
	Residuals	447	117.28	0.26	0.80		
	Total	482	146.96	1.00			
Unifrac	Health status	5	2.99	0.60	7.75	0.065	0.001
	Sex	1	0.35	0.35	4.49	0.008	0.001
	Age	1	0.89	0.89	11.52	0.019	0.001
	Participant	28	7.35	0.26	3.40	0.159	0.001
	Residuals	447	34.53	0.08	0.75		
	Total	482	46.11	1.00			
Weighted unifrac	Health status	5	0.81	0.16	6.40	0.055	0.001
	Sex	1	0.09	0.09	3.57	0.006	0.006
	Age	1	0.47	0.47	18.76	0.033	0.001
	Participant	28	1.91	0.07	2.71	0.132	0.001
	Residuals	447	11.24	0.03	0.77		
	Total	482	14.52	1.00			

**FIG 3:**
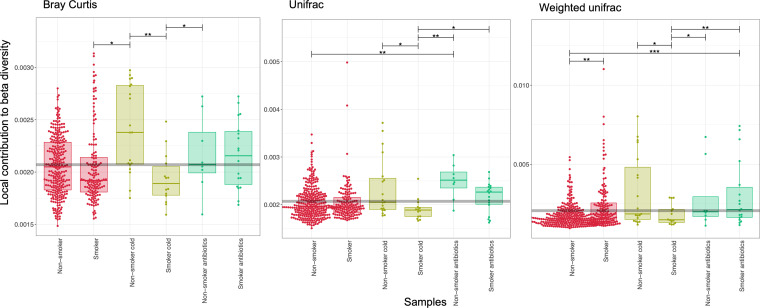
Beta diversity measures using Bray-Curtis (counts), unweighted unifrac (phylogenetic distance), and weighted unifrac (phylogenetic distance weighted by abundance counts) of the oropharyngeal microbiota of nonsmokers and smokers when healthy, expressing common cold symptoms, or taking antibiotics. Note the difference in scale. Means are given as horizontal gray lines. The lines connect two categories if the values are significant per an ANOVA, with one within subject factor accounting for the participant IDs.

The local contribution to beta diversity (LCBD) is a measure providing insight into the ecological uniqueness (i.e., unusual species combinations) of a sample. Based on the abundance count (Bray Curtis) (Fig. 3), it was evident that nonsmokers with cold symptoms had significantly higher degrees of ecological uniqueness, compared to smokers and smokers with colds. When considering phylogenetic distances only (unifrac) (Fig. 3), nonsmokers on antibiotic treatments had particularly high LCBD values, which are indicative of relatively high degrees of ecological uniqueness. Using both abundances and phylogenetic distances, this pattern disappeared (weighted unifrac) (Fig. 3), but nonsmokers experiencing cold symptoms generally showed the greatest variability in LCBD.

We subsequently used a subset analysis (BVSTEP routine) to determine which OTUs were responsible for the changes in beta diversity, comparing samples between smokers and nonsmokers in three groups: healthy participants, participants exhibiting cold symptoms, and participants on antibiotics. To do so, we collapsed the abundance table to the minimum set of OTUs explaining differences in community composition between groups. This resulted in a reduced-order abundance table that correlated highly with the full OTU table (Supporting information Table S2; Fig. S1). There were 24 OTUs associated with differences in community composition between healthy smokers and nonsmokers, 15 OTUs associated with nonsmokers and smokers experiencing cold symptoms, and 17 OTUs driving differences in nonsmokers and smokers on antibiotics. These three groups shared seven OTUs belonging to different genera. For further information on the relationships between features, the microbial communities (based on abundance tables), and the sources of variation (age, years smoking, and cigarettes smoked/week), refer to Fig. S6.

**FIG 4:**
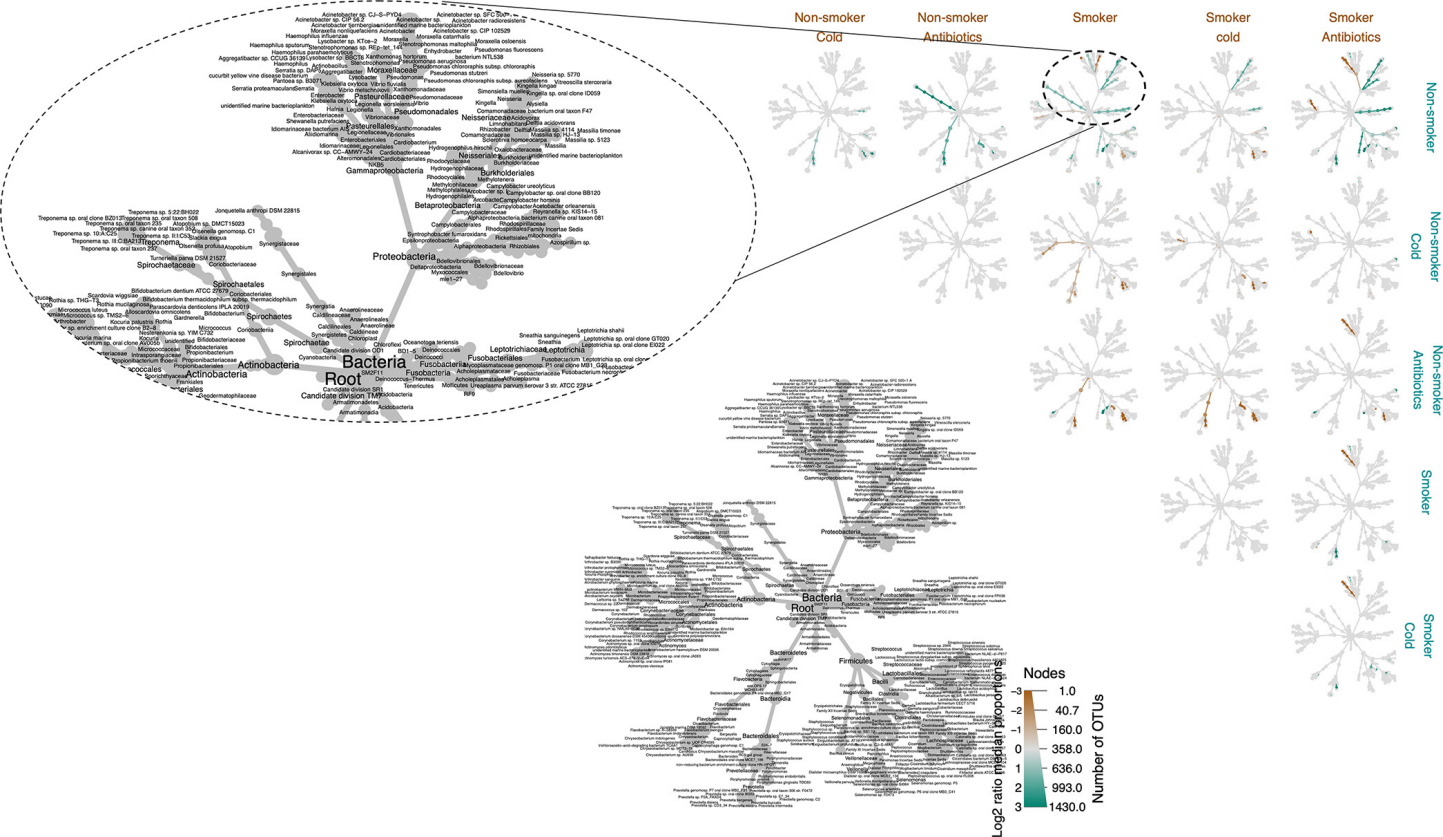
Heat tree matrix comparing the OTUs of the bacterial core of the oropharyngeal microbiota of smokers and nonsmokers when healthy, expressing common cold symptoms, or taking antibiotics. The lower left-hand side diagram shows the phylogeny of the pooled data set, and the sizes of the circles associated with different taxa indicate their relative abundances. The brown and cyan colors indicate significant differences across pairwise abundances, with the color indicating in which group the abundances were greater (with deeper colors indicating higher abundances). The gray color represents no significant difference in relative abundance. Refer to a high resolution rendering in Fig. S5 in the supplementary materials.

A heat tree analysis revealed OTUs in significantly different abundances across all samples, based on smokers, nonsmokers, smokers and nonsmokers expressing common cold symptoms, and smokers and nonsmokers on antibiotics (Fig. 4) (refer to a high resolution rendering in Fig. S5). A heat tree analysis of healthy smokers and healthy nonsmokers showed greater a relative abundance of Fusobacteriaceaes, Neisseriales, and Spirochaetidea in nonsmokers and a greater abundance of Actinobacteria in smokers.

Healthy nonsmokers had a greater abundance of Bacteroidales and Fusobacteria, compared to nonsmokers with colds. Moreover, healthy nonsmokers showed a greater abundance of Spirochaetidae and Porphyromonadacea, compared to nonsmokers on antibiotics. Interestingly, there were no differences in abundance between nonsmokers with colds and nonsmokers on antibiotics. Healthy smokers and smokers with cold symptoms showed no differences in microbial community abundance, whereas smokers on antibiotics had greater abundance of Klebsiella and Enterobacter but lower *Porphyromonas*, compared to healthy smokers.

**FIG 5:**
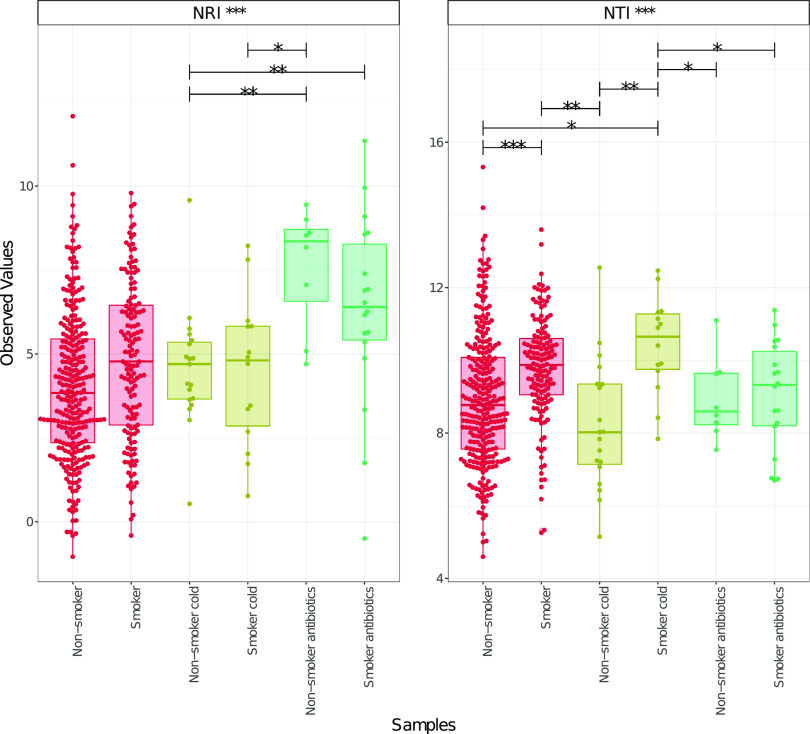
Nearest-taxon-index (NTI) and nearest-relative-index (NRI), considering the presence/absence of OTUs in the microbiota samples of smokers and nonsmokers when healthy, expressing common cold symptoms, or taking antibiotics. Note the difference in scale. The lines connect two categories if the values are significant per an ANOVA, with one within subject factor accounting for the participant IDs.

Next, we explored whether the microbial assemblage composition was driven by the host environment or by competition via the use of the nearest-relative-index (NRI) and the nearest-taxon-index (NTI). Values greater than 2 indicate strong phylogenetic clustering (driven by environmental filtering), whereas those less than 2 indicate phylogenetic overdispersion (indicating the role of ecological interactions, rather than the environment, in shaping the communities). Our analysis suggested that environmental filtering (rather than competition) was the process driving community composition in the oropharynx (Fig. 5). Moreover, for NTI, environmental filtering was greater in smokers than in nonsmokers. It is worth considering that NRI reflects phylogenetic clustering in a broad sense (considering the whole phylogenetic tree), with the negative values representing an evenly spread community. Non-smokers on antibiotics had the greatest NRI values, suggestive of the highest degree of environmental filtering, when considering the phylogenetic tree as a whole. NTI focuses on the tips of the tree, with positive values indicating that species co-occur with more closely related species than expected and negative values indicating that closely related species do not co-occur. Evidently, when showing cold symptoms, environmental filtering was significantly higher in smokers than in nonsmokers, suggesting that more species co-occurred with closely related species. In general, environmental filtering was greater in relation to closely-related species (NRI), compared to when accounting for the whole phylogenetic tree (NTI).

For each participant, the core microbiota was identified to infer interaction matrices. These were used to determine the dynamical properties of communities by inferring the Jacobian matrix and its dominant eigenvalue, indicating that all communities were locally stable. We subsequently determined whether the local stability of the smokers and nonsmokers’ oropharyngeal microbiota differed by comparing their dominant eigenvalues (nonsmokers mean = −5.908, SD = +3.537; smokers mean = −6.953, SD = +2.989). An analysis of variance (ANOVA) indicated no difference between them (F_1,21_ = 0.495, *P* = 0.490).

To determine whether they were globally stable (i.e., permanent), interaction matrices were then tested for the existence of an average Lyapunov function. Out of the 15 inferred interaction networks from nonsmokers, 6 (40%) were globally stable. Out of the 8 smoker networks, 4 (50%) were globally stable. Thus, our results provide no evidence for differences in local or global stability between smokers and nonsmokers.

With the exception of one nonsmoker interaction network, all smoker (*n* = 8) and nonsmoker (*n* = 14) communities were locally stable after a new species was added to the Jacobian matrix, indicative of resistance to species invasion, irrespective of smoking status.

## DISCUSSION

The oral microbiota plays a vital role in the maintenance of oral health, and dysbiosis is associated with a number of diseases, including periodontal disease (43, 44). Findings of studies investigating the effects of smoking on the oral microbiota have been inconsistent, with some reporting changes in community composition that are associated with varying taxa (5, 20, 45, 46), while others reported no association between the oral microbiota and smoking (47–49). Variability in the definition of smoking, sample site selection, methodology, study population, and inclusion/exclusion of samples based on preconditions are likely to account for at least some of these inconsistencies. Here, we collected longitudinal samples of the oropharyngeal microbiota of 30 healthy participants (18 nonsmokers and 12 smokers), using a standardized sampling approach by sampling the same site and using the same methodology (as described above) in the same participants and study population, avoiding the issues noted above.

Smoking constitutes a press perturbation that could result in changes in the richness, composition, and abundance of the microbiota. As hypothesized, in our results, smoking decreased alpha diversity in line with that reported by other studies (50, 51), similar to antibiotic use, which we consider to be a short-term pulse perturbation (52, 53). Our results suggest that this process results in lower diversity in communities undergoing a simultaneous, significant press perturbation (i.e., smoking). Fewer niches being available due to a relatively more hostile microclimate may result in a lower species richness and a loss of protection by mutualistic species and, thus, disease (21, 54). However, others (6) have found that smokers had increased alpha diversity (Shannon diversity), compared to nonsmokers, arguing that smoking decreases the niche saturation abilities of a community that selects for a specific group of organisms.

As hypothesized, our study provided evidence that press perturbations, such as smoking, impacted beta diversity, even within the context of short-duration pulse perturbations (cold or antibiotic use). Our results showed that the seven OTUs that were responsible for the changes in the beta diversity patterns were shared across smokers and nonsmokers, regardless of their health status. The effect of the short-term pulse perturbations in the form of cold and antibiotic use was evident when measuring the local contribution to beta diversity (LCBD), which indicated a high uniqueness of the bacterial community composition. Our analysis showed that nonsmokers exhibiting cold symptoms and participants on antibiotics (irrespective of smoking status) displayed the greatest LCBD values. Colds only affected undisturbed communities (i.e., nonsmokers), whereas sufficiently large pulse perturbations (antibiotic treatment) impacted smokers and nonsmokers (i.e., disturbed and undisturbed communities) equally, as evidenced by the alpha diversity values becoming more similar between smokers and nonsmokers. This suggests that disturbed communities become more similar to one another, irrespective of smoking status.

High values of the nearest-relative-index (NRI) and nearest-taxon-index (NTI) provide evidence that these oropharyngeal communities are dominated by environmental filtering. This may be related to nutritional, spatial, and metabolic factors selecting for species that possess suitable traits to exist within specific conditions (55). As hypothesized, smokers show greater environmental filtering, compared to nonsmokers, and participants on antibiotic treatment show the greatest degree of environmental filtering, irrespective of smoking status, suggesting that these forms of environmental perturbations are strong enough to override the importance of ecological interactions in shaping the microbial communities. Smoking is likely to affect the microenvironment, specifically, the oxygen, pH, and acid production, thereby resulting in the selection of specific microbial community members of microaerophilic and anaerobic bacteria that can dominate due to the lower oxygenation (6, 56).

Studies have shown that more diverse and complex communities tend to be generally more resistant to perturbations (37). Contrary to our expectations, stability models based on our data indicated that local and global stability did not differ between smokers and nonsmokers, suggesting that microbial communities of smokers are resistant and resilient to small and large perturbations. Moreover, our results indicated no differences in invasibility between smokers and nonsmokers when invasibility was measured as instability.

The human microbiota is often found to be stable and generally able to maintain homeostasis, even in the face of perturbations (57). Other authors have argued that the oral cavity is characterized by an even greater degree of stability, compared to that of other body sites (58). The heterogeneity of the oral cavity increases niche space, in which many microbial community members live on surfaces in biofilms, which likely allows them to survive adverse conditions, such as smoking and antibiotic exposure (24, 51, 59). Moreover, others have found that even upon smoking cessation, the differences in microbial community composition generally persisted over the time period of this study, suggesting that smoking can introduce an alternative but stable state (22).

The microbiota is not just a passive passenger; its modification can play a key role in contributing to or causing many pathophysiological processes (21, 42, 60). Others have shown that smoking creates pathogen-rich and commensal-poor communities (5, 6). Similar to the findings of others, there were decreases in the relative abundance of *Prevotella* and *Neisseria* spp. (61, 62) and an increased relative abundance of *Veillonella* spp. in smokers, compared to nonsmokers (50). These differences may be associated with differences in oxygen tension, with smoking promoting the presence of anaerobes (63). Overall, we found no evidence suggesting that smoking communities in this particular group of participants were characterized by pathogen-rich and commensal-poor species. It may be worth noting, however, that the excess of Gram-negative Klebsiella and Enterobacter in smokers on antibiotics are increasingly seen in aspiration pneumonia (64), which could suggest that smoking/antibiotics are possible risk factors for aspiration pneumonia caused by these organisms in older age groups.

In conclusion, our study provides evidence that microbiota changes with smoking are likely a combination of parts of the microbiota not surviving the direct toxicity of cigarette smoke and its indirect consequences via the associated changes in the immune system and cellular processes, in general. This manifested in the analyses as the importance of environmental filtering in the structuring of the microbial communities. This suggests that environmental filtering dictates which OTUs may be present in the community, resulting in the perturbed communities’ final, alternative states. Even though the oropharyngeal microbiota of smokers is reduced in terms of diversity, compared to that of nonsmokers, it is ecologically no less stable or more invasible. Therefore, the increased risk of infectious diseases in smokers is not necessarily facilitated by the effect of smoking on ecological changes in the composition or function of the oropharyngeal microbiota but is more likely due to its effects on the local and systemic immune system (65), which may alter the susceptibility to contract an infection and the course of disease (66, 67). A next step to support the findings from these analyses would be to conduct experiments or generate empirical data that investigate the stability and invasibility of microbiota in response to cigarette smoke, cold-symptoms, or antibiotic disturbances.

## MATERIALS AND METHODS

### Participants and sample collection.

30 participants (18 nonsmokers and 12 smokers) were recruited for this study, representing both genders (21 females, 9 males) and ranging in age from 18 to 40 (mean = 25.18 years, SD = 6.71). 10 of the females were smokers (smoking on average 32.25 [SD = 25.76] cigarettes per week, having smoked for 5.1 [SD = 3.70] years on average), and 2 of the male participants were smokers (smoking on average 43.14 [SD = 24.12] cigarettes per week, having smoked for 9.5 [SD = 9.19] years on average). Further information on sample sizes is provided in Table S1.

All participants were healthy (without illnesses or undergoing medical treatment), not on any long-term medication, and had not stopped smoking recently. The study was approved by the University of Glasgow Ethics Committee (Ethics Applications 2012107 and 200140021).

Participants were provided with Sigma Transwabs swabs in liquid amies (Medical Wire Ltd., United Kingdom) for bacterial detection, and samples were taken from the tonsils and the posterior wall of the tonsil, as suggested in the literature (68). Participants were asked to take a swab once weekly, early in the morning, prior to brushing their teeth and having breakfast, as well as to keep a diary providing information about their health status, self-reporting any changes there. Samples were collected weekly, and bacterial swabs were processed, after being kept on ice, within 2 h after collection. Sampling occurred in Glasgow (United Kingdom) for the nonsmokers from January until May of 2013, and sampling recommenced from September until December of 2013. The sampling period for the smokers took place from November of 2014 to June of 2015, giving a sampling period of 30 weeks. Staffing constraints did not allow for the sampling of smokers and nonsmokers within exactly the same time period. While we recognize this issue, we argue it unlikely that the differences observed between the smokers and nonsmokers are driven solely by annual fluctuations in microbiota.

### DNA extractions.

DNA was extracted using a QIAamp DNA Minikit (Qiagen Ltd., United Kingdom), following the manufacturer’s instructions. The extracted DNA was quantified using a Qubit 2.0 (Thermo Fisher Scientific, Q32866) and a Picogreen HS DNA Assay (Invitogen Ltd., United Kingdom). 5 μL of DNA were mixed with 2 μL of loading dye on a 1% agarose gel (1 g agarose to 100 mL TBE), along with a 1 kb Invitrogen DNA ladder, and run at 100 V for 60 min to check purity, and they were subsequently stored at –20°C until required (69). The details on processing the samples are given in Chapter 2 of http://theses.gla.ac.uk/8163/, and they have also been described in Rani Ram (2017) (70).

A clone library was prepared using an Invitrogen Topo-Seq Kit (Invitrogen Ltd., United Kingdom) as a quality control step to produce a mock community for future Illumina MiSeq runs. The purpose of the mock community was to act as a positive control for each MiSeq run so as to ensure that the correct sequences were being sequenced. To reduce PCR biases, all of the template DNA was diluted to the same concentration, and the minimum number of cycles was used in each PCR run to reduce nonspecific binding, with appropriate controls in place. A positive control (DNA from the mock community) and a negative control (for each different reverse barcode, using nuclease free water) was set up for each PCR run. To ensure that the source of bacterial sequences was not the swab itself or the DNA isolation reagents, PCR was performed on DNA isolated from an unused swab. To confirm that the PCR reagents were not sources of bacterial sequences, a PCR of the no-template extraction control was also performed.

### Bioinformatics.

Abundance tables were obtained by constructing OTUs (a proxy for species) as previously described (42) and as follows. The trimming and filtering of the paired-end sequencing reads was performed using Sickle (version 1.2) by applying a sliding window approach and trimming the regions where the average base quality dropped below 20 (71). This applied a 10 bp length threshold to discard reads that fell below this length. BayesHammer (72) was used from the SPAdes assembler (version 2.5) to error correct the paired-end reads, and this was followed by PANDAseq (version 2.4) with a minimum overlap of 50 bp to assemble the forward and reverse reads into a single sequence that spanned the entire V1-V2 region (73). The above choice of software showed a reduction in substitution errors by 77 to 98%, with an average of 93.2% for the MiSeq data sets (74). UPARSE (version 7.0.1001) was used for OTU construction (75). The approach pools together the reads from different samples and adds barcodes to keep an account of the samples from which these reads originate. The reads are then dereplicated and sorted by decreasing abundance, discarding singletons. The reads were clustered based on 97% similarity, discarding any reads that were shorter than 32 bp. The original barcoded reads were then matched against OTUs with 97% similarity (a proxy for species-level separation) to generate OTU tables for different samples. The representative OTUs were then taxonomically classified against the RDP database, using the standalone RDP classifier (version 2.6) (76). The phylogenetic distances between OTUs were produced by first using MAFFT (version 7.040) (77) to align the OTUs against each other and then by using FastTree (version 2.1.7) on these alignments to generate an approximately maximum likelihood phylogenetic tree (78). Potentially contaminating amplicon sequence variants were identified and removed using the decontam package in R (79). The OTU table, phylogenetic tree, taxonomic information, and metadata were then used in a statistical analysis. Chimeras were removed using two approaches: an external Chimera Slayer Gold database and a *de novo* approach in which the abundant reads served as a reference database. Traditional pipelines were modified to get the optimum accuracy for amplicons, based on benchmarking studies (73, 80, 81). Subsequently, the OTU table, phylogenetic tree, taxonomic information, and metadata were used in a multivariate statistical analysis. While the *de novo* chimera removal step removes reads that have chimeric models that are built from more abundant reads, a few chimeras may be missed, especially if they have parents that are absent from the reads or are present in low abundance. To deal with that, we used a reference-based chimera filtering step from a Gold database (http://drive5.com/uchime/uchime_download.html) that is derived from the ChimeraSlayer reference database in the Broad Microbiome Utilities (http://microbiomeutil.sourceforge.net/).

### Statistical analysis.

Statistical analyses were performed in R, using the tables and data that were generated as well as the metadata associated with the study. All samples that contained less than 5,000 reads were discarded in the analysis to allow for the comparison of all samples with sufficient statistical power.

For the community analysis (including the alpha and beta diversity analyses), we used the vegan package (82). For the alpha diversity measures, which provided us with an insight into the changes in richness and evenness with smoking, antibiotic, and cold status, we calculated: OTU richness (the estimated number of OTUs per sample), Shannon entropy (a commonly used index by which to measure the richness and evenness of a community within a sample), and Simpson diversity, which takes both sample richness and evenness into account but is less sensitive to species richness, using Renyi-Hill numbers (83). The alpha diversity measures were calculated after rarefying the abundance table to the minimum library size by using rarefaction curves that were obtained for each participant to approximate the OTUs that were detected as a function of the sequencing depth. As a result, the samples were rarefied to the minimum number of reads (5,118) to test for alpha diversity, and the relative abundance of taxa for each sample was calculated by dividing the read counts of a taxon by the sample size. This value ranges from 0 to 1. Rarefaction curves can be found in Rani Ram (2017) (70). The data were analyzed using an ANOVA with Tukey’s *post hoc* test, after checking the assumptions (normality, heterogeneity of variance, and independence). To adjust for patient IDs, all of the pairwise statistics were done using one within subject factor in anova, as aov(value ~ Groups + Error(ParticipantID/Groups)), by incorporating Error().

In order to investigate the multivariate homogeneity of the group dispersion (i.e., how variable the groups are) between multiple conditions (i.e., smoking, antibiotic, and cold status) we used vegan’s betadisper() function to calculate three different distance measures: Bray-Curtis (which considers the species abundance count), unweighted unifrac (which considers the phylogenetic distance between the branch lengths of OTUs that are observed in different samples without taking into account the abundances) and weighted unifrac (unweighted unifrac distance, weighted by the abundances of the OTUs), using the metamds() function in vegan. The distances between objects and group centroids are handled by reducing the original distances to principal coordinates and subsequently performing an ANOVA on them, according to a four-factor design, including health status (six levels: nonsmoker, smoker, nonsmoker with cold, smoker with cold, nonsmoker on antibiotics, smoker on antibiotics), sex (two levels: male, female), age (continuous), and participant (30 levels). We used vegan’s adonis() function for an analysis of variance using distance matrices to partition sources of variation. This function will be referred to as PERMANOVA, and it fits linear models to distance matrices and uses a permutation test with pseudo-F ratios (84).

To measure beta diversity, we performed a local contribution to beta diversity (LCBD) analysis (85). In the context of this longitudinal study, LCBD provides a means by which to show the difference of the microbial community structure from a single sample, based on the average of all samples (with higher LCBD values representing communities with greater beta diversity values or outliers). We used the LCBD.comp() function from the adespatial package (86) to calculate three different measures: Hellinger distance (abundances), unweighted unifrac (phylogenetic distance), and weighted unifrac (phylogenetic distance weighted by abundance) dissimilarities. LCBD gives the sample-wise local contributions to beta diversity, which are derived from the proportions of the total beta diversity.

We subsequently used the BVSTEP routine to complement the beta diversity analysis, as it is an algorithm that allows for the identification of the OTUs that are causing the major shifts in beta diversity. The BVSTEP algorithm (87) searches for the highest correlation between the dissimilarities of fixed and variable multivariate data sets using the bvStep() function from the sinkr package, using a permutation of the 2^n^-1 features in the data set (i.e., OTUs) (88). Testing all feature combinations is unrealistic and computationally intractable when the species richness in the data set is high. To deal with this, we used the abundance table with the 1,000 most abundant OTUs to best correlate with the overall similarities in the features (i.e., grouping factors, smoking, cold, and antibiotic status), given all of the OTUs, assuming that the most abundant species may play a significant role.

To determine whether the phylogenetic community structure in each sample was driven by competition among taxa or by strong environmental pressure (i.e., the host environment) we calculated the mean-nearest-taxon-distance (MNTD, using the mntd() function), nearest-taxon-index (NTI, using the ses.mntd() function), mean-phylogenetic-diversity (MPD, using the mpd() function) and nearest-relative-index (NRI, using the ses.mpd() function) in the picante package in R (89). NTI and NRI are the negative outputs from the ses.mntd() and ses.mpd() functions, respectively.

NTI and NRI quantify the number of standard deviations that the observed MNTD and MPD values, respectively, is from the mean of the null distribution (999 randomizations using null.model = “richness”) in the ses.mntd() and ses.mpd() functions, only considering the taxa as present/absent, without taking their abundances into account. Based on recommendations, we used the top 1,000 most abundant OTUs for the calculation of these measures (90).

Subset regression is a model selection approach that provides evidence for the important factors that are associated with changes in the microbial community composition, including the effects of different disturbances (e.g., smoking, cold, and antibiotic status), thereby providing further support for the above analysis. Subset regression was performed against different microbiota metrics, testing all possible combinations of explanatory variables and subsequently selecting the best model, according to statistical criteria, based on recommendations (91). The code is available at (http://www.sthda.com/english/articles/37-model-selection-essentials-in-r/155-best-subsets-regression-essentials-in-r/) (91). We used the regsubsets() function from the leaps package (92) to identify the best models of different sizes by specifying the option nvmax set to the maximum number of predictors to be incorporated in the model. After having obtained the best subsets, the k-fold cross-validation consisted of first dividing the data into k subsets, of which each subset (10%) served successively as test data and the remaining subset (90%) as training data, using a custom function that utilized the train() function from the caret package (93). The average cross-validation error was calculated as the model prediction error. We used the tab_model() function from the sjPlot package to obtain the statistics for each model (94).

There has been no single standardized approach by which to identify the core microbiota, with most studies reporting the core based on the presence/absence of taxa at specific threshold values of abundance and prevalence (95). Here, we defined the core microbiota by setting a detection threshold of 0.001 (i.e., 0.1% compositional abundance threshold in a sample) and a prevalence of the OTUs to be found in samples between 85 and 92% (depending on the total sample size available for each longitudinal data set) for each participant of this study, following advice given in Shetty (96) and using the microbiota package (97).

We subsequently used the heat_tree_matrix() function in the metacoder package (98) to detect differences in the relative abundances in the core OTUs (based on the log_2_ median ratio) between participants, using a Wilcoxon test.

### Local and global stability and invasibility.

Here, we refer to stability in the context of ecological community dynamics, defined broadly as the ability of an ecosystem to persist through perturbations, which is influenced by changes in the interactions between different species. Understanding under which conditions the species in a community coexist in the long term is a central question in ecology that can be addressed by investigating local and global stability. Local stability is a measure of the persistence of communities subjected to small perturbations, whereas global stability (also referred to as permanence) refers to a global property of a community that only requires the densities of species to increase when they are rare (99, 100). To address the question of whether the potentially reduced stability of smokers’ microbiotas could be involved in pathogen invasions, we characterized the ecological stability of the microbiotas of both smokers and nonsmokers by using two methods, namely, local and global stability analysis, working under the assumption that microbiotas, including the pharyngeal microbiotas, are stable over time (45, 101, 102).

The calculation of these stability measures requires the inference of the microbial core for each participant as described above; we used the limits() function in the seqtime package (103, 104), which utilizes time-series data, to infer the microbial interaction matrices that corresponded to the classical generalized Lotka-Volterra dynamics through deterministic model fitting. We only selected participants for which we had at least nine samples (after discussion on sample size and network inference with the package authors). This resulted in estimated interaction matrices for 15 nonsmokers and 8 smokers.

Interaction matrices can be used to determine the dynamical properties of communities by inferring the Jacobian matrix and its dominant eigenvalue, where the equilibrium is locally stable if all of the eigenvalues of the interaction matrix have negative real parts (100, 105, 106). For each inferred interaction matrix, we computed the dominant eigenvalue (the most positive eigenvalue) to evaluate local stability.

Local stability applies only to small perturbations, and its ecological relevance has been debated (100, 107, 108). A dynamical system is globally stable if it does not experience extinction following arbitrarily large stochastic perturbations. Communities are defined as globally stable if an average Lyapunov function exists near the boundary of the state space and if the system is dissipative (109), meaning that even if a system experiences a large perturbation, it will return to some form of equilibrium without resulting in species extinction. To determine whether the interaction matrices were globally stable, we tested our interaction matrices for the existence of an average Lyapunov function using (110) “sufficient condition”, as laid out prior (111), solving the problem as a linear programming problem in MATLAB. Here, we calculated both measures of stability for the interaction matrix estimated for the core communities of each participant to determine if those in smokers were less stable, globally and locally, compared to those of nonsmokers (110, 111).

To test whether the microbial communities in smokers were more invasible, for each participant, we recomputed the equilibrium point of the Jacobian matrix by adding a new species to the community (where all of the rows and columns of the “invader” OTU were zero) and calculating the dominant eigenvalue of this augmented interaction matrix. If stable, then the species were at equilibrium and were not increasing in abundance (i.e., the community was not invasible). If unstable, then the community is invasible, with the invader increasing in abundance.

### Data availability.

The following data: otu_table.biom, otus.fasta, otus.tre, and meta_data.csv file are available on GitHub (https://github.com/umerijaz/pharynxmicrobiome). The scripts are available as part of microbiomeSeq (https://userweb.eng.gla.ac.uk/umer.ijaz/projects/microbiomeSeq_Tutorial.html) and at https://userweb.eng.gla.ac.uk/umer.ijaz/bioinformatics/ecological.html.
