# Supplementary material for: The Effects of Smoking on Human Pharynx Microbiota Composition and Stability
Source: Microbiol Spectr. 2023 Feb 14;11(2):e02166-21. doi: 10.1128/spectrum.02166-21 (PMC10101099; doi:10.1128/spectrum.02166-21)
Supplement: Supplemental file 2 — Supplemental material. Download spectrum.02166-21-s0002.pdf, PDF file, 4.2 MB [file spectrum.02166-21-s0002.pdf]

SUPPLEMENTAL MATERIAL

Table S1. Overview of available samples

			Smoker			Non-smoker		
			**Healthy**	**Cold**	**Antibiotics**	**Healthy**	**Cold**	**Antibiotics**
Number of samples	Female		98	14	18	169	14	4
	Male		47			110	5	4
Age (years)	Female	Median	19.00	19.00	19.00	21.00		
		Mean	21.75	19.00	19.00	22.91		
	Male	Median	36.50			20.00		
		Mean	36.50			21.57		
Cigarettes smoked per week	Female	Median	25.00	35.00	60.00			
		Mean	28.88	35.00	60.00			
	Male	Median	30.00					
		Mean	40.00					
Years smoking	Female	Median	3.50	5.00	5.00			
		Mean	5.13	5.00	5.00			
	Male	Median	17.50					
		Mean	17.50					

Table S1. Overview of available samples

[Excel document]

Table S2. Subset analysis from BVSTEP routine listing top subsets with highest correlation with the full OTU table considering Bray-Curtis distance based on comparisons between smokers and non-smokers, smokers and non-smokers with cold symptoms and smokers and non-smokers on antibiotics.

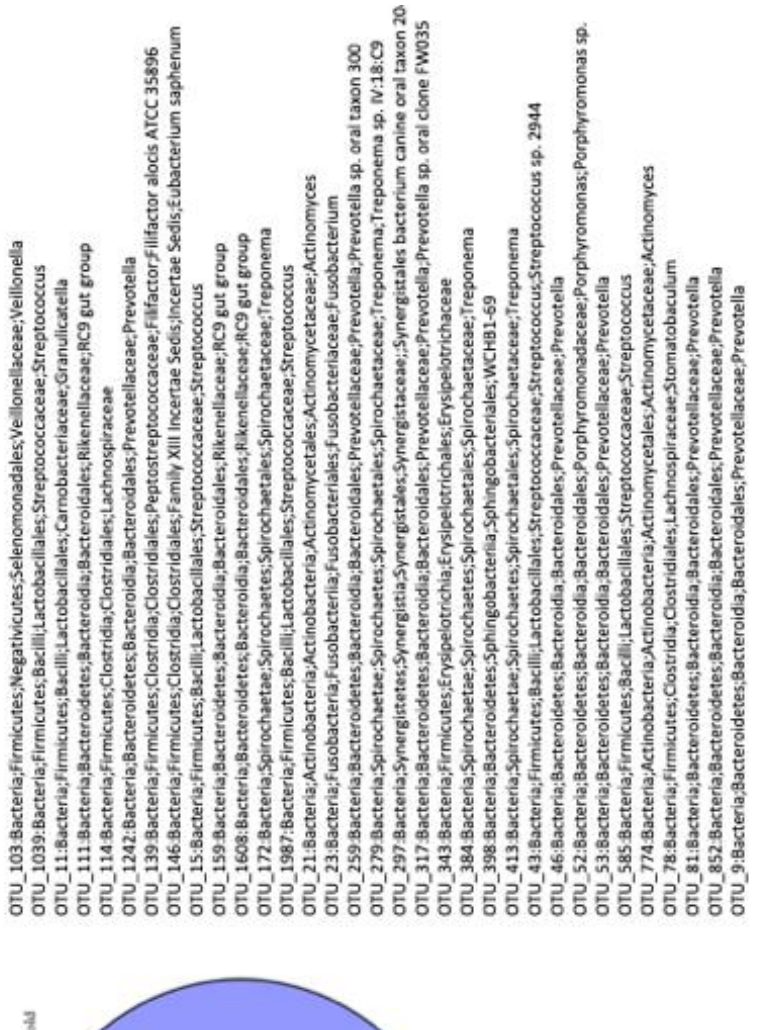

Figure S1. Venn diagram showing results of the subset analysis from the BVSTEP routine showing OTUs with highest correlation to the full OTU table considering Bray-Curtis distance based on comparisons between smokers and non-smokers (yellow), smokers and non-smokers with cold symptoms (blue) and smokers and non-smokers on antibiotics (red).

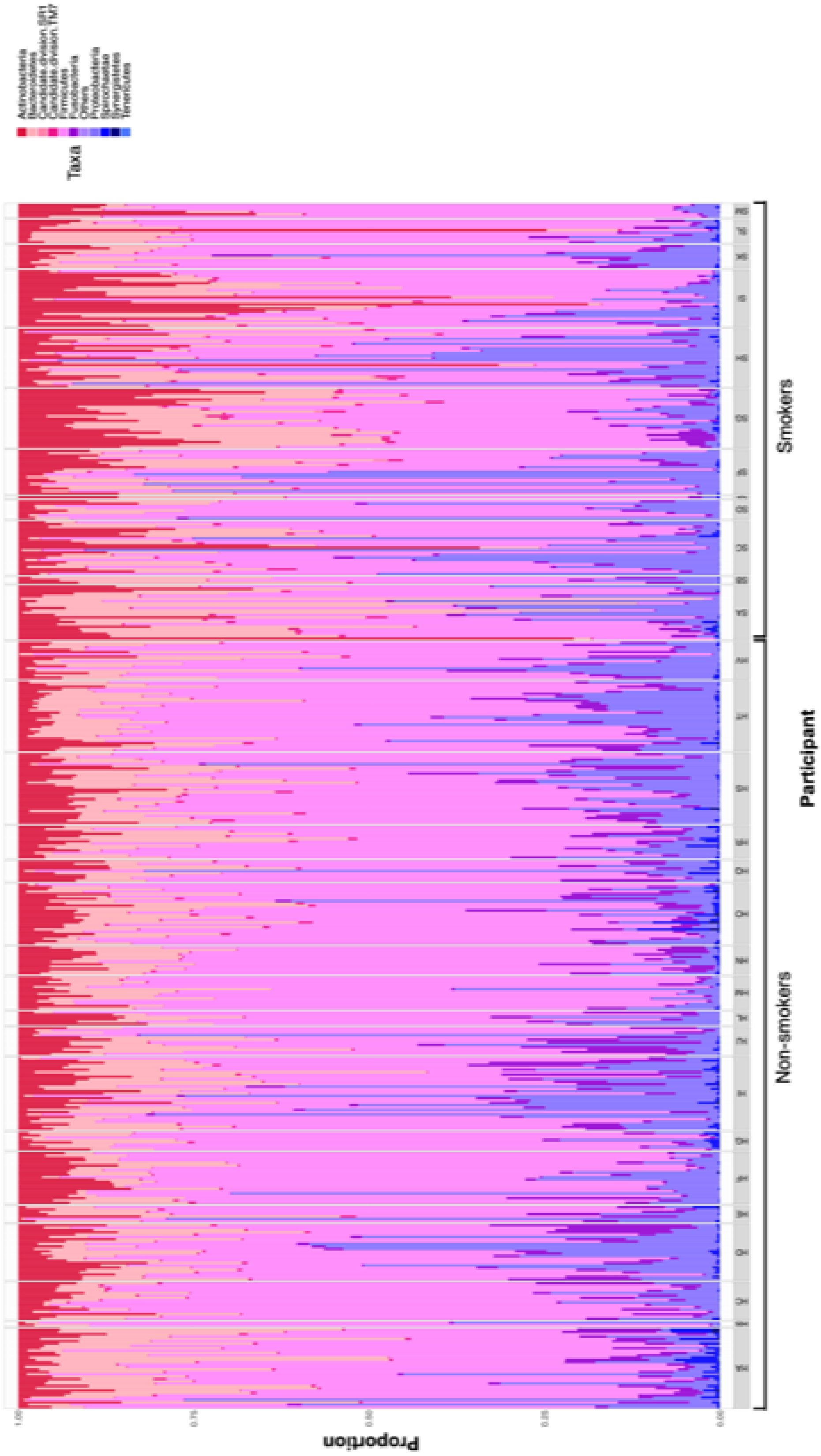

Figure S2. Relative abundance (%) of the ten dominant phyla for smokers and non-smokers for each week over the sampling period.

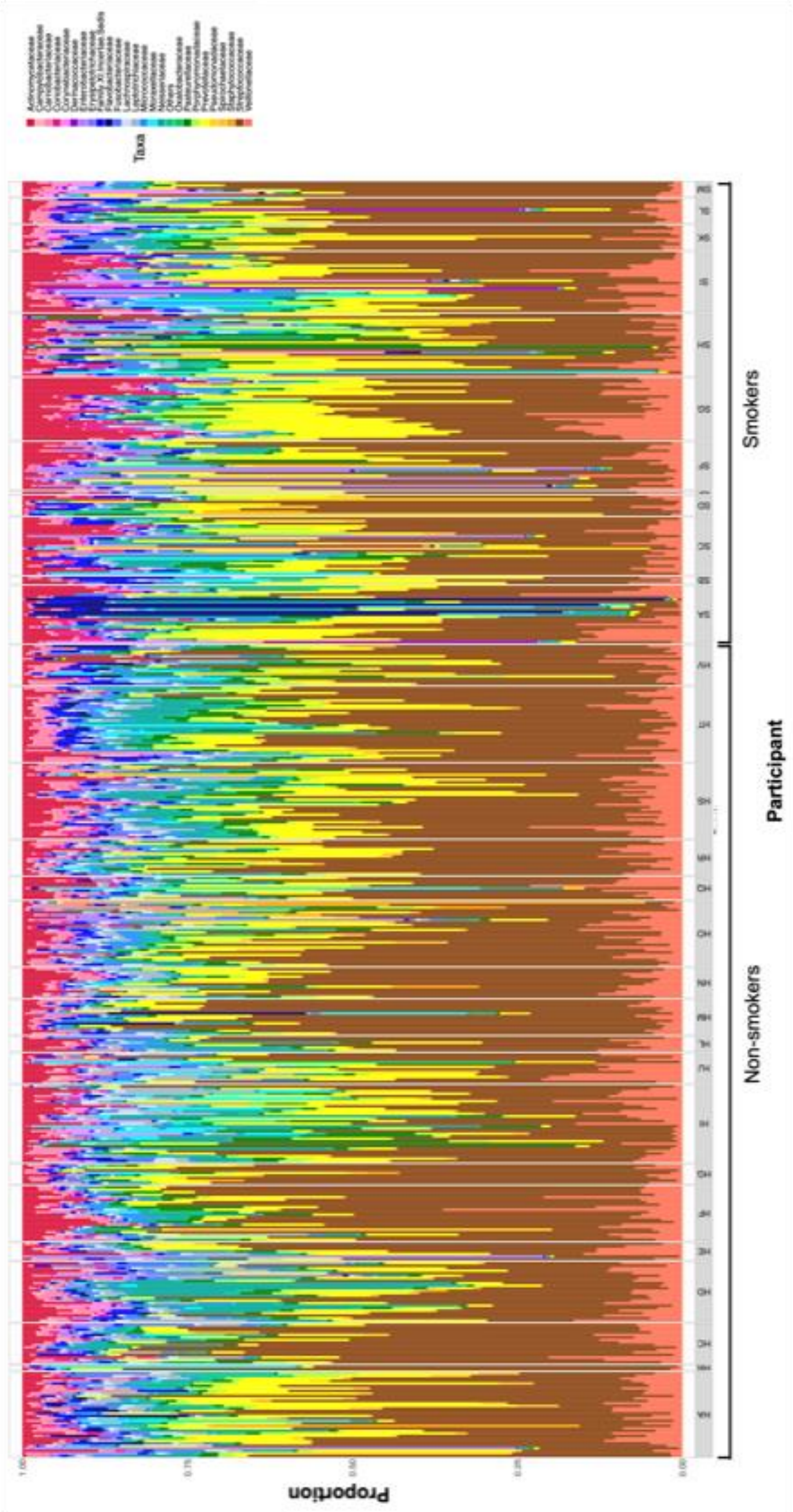

Figure S3. Relative abundance (%) of the 25 dominant families for smokers and non-smokers for each week over the sampling period.

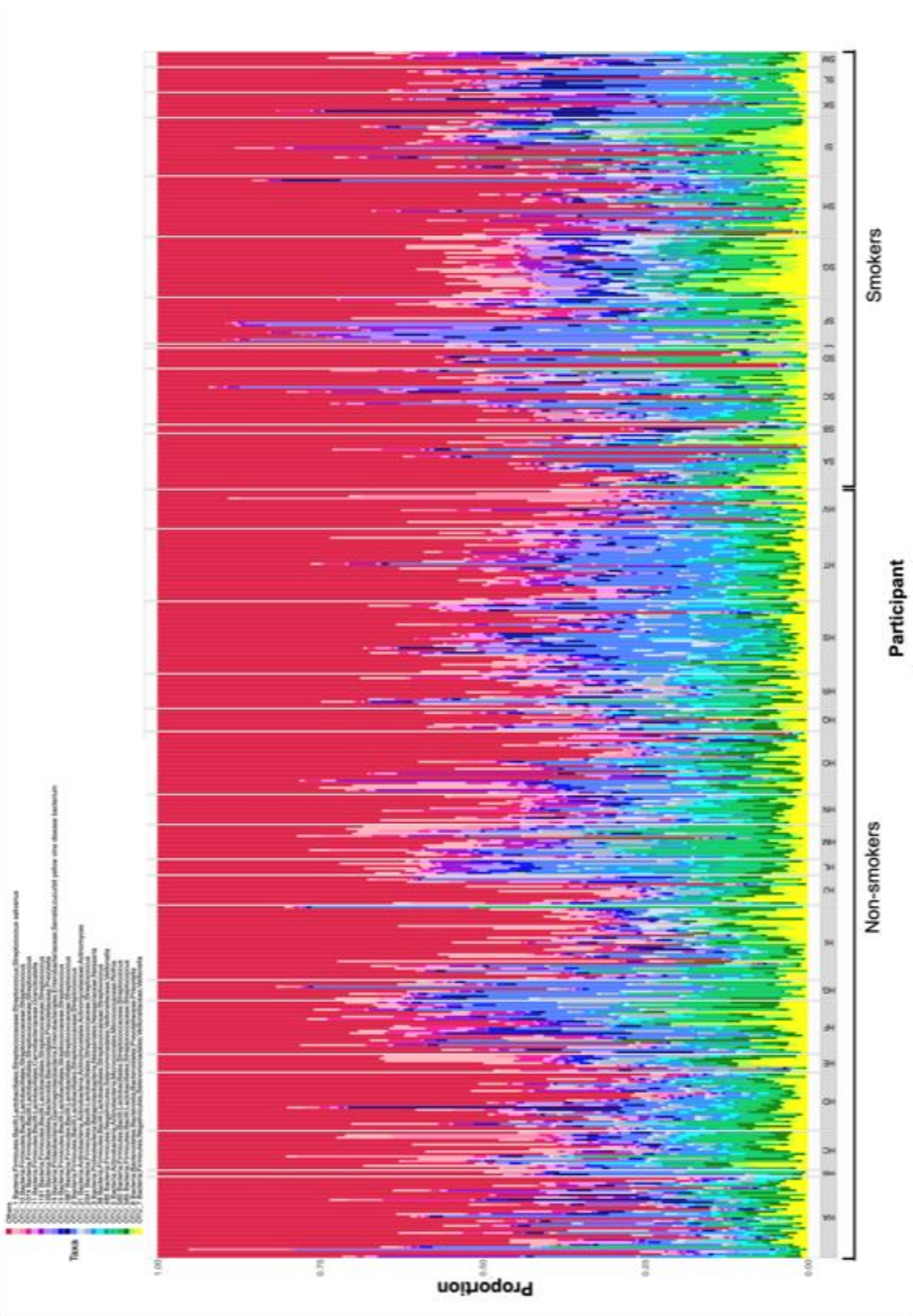

Figure S4. Relative abundance (%) of the 20 dominant OTUs for smokers and non-smokers for each week over the sampling period.

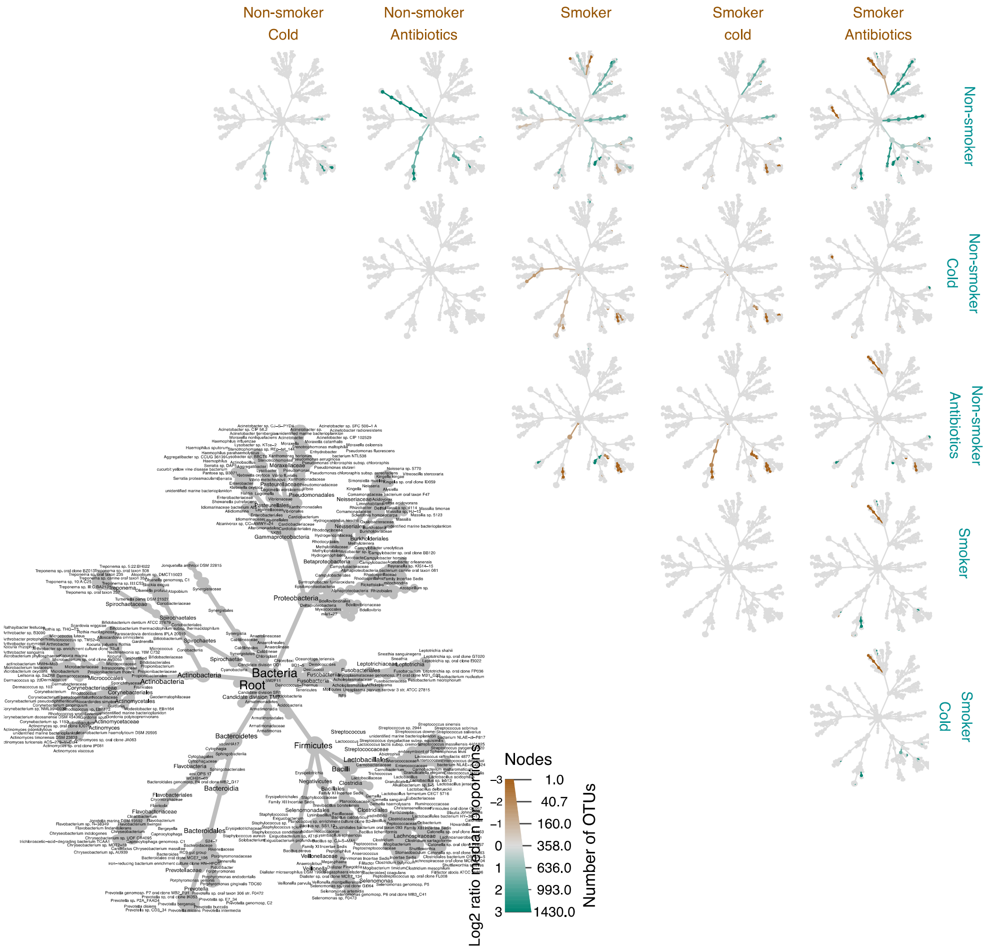

Figure S5. Vector format: heat tree matrix comparing OTUs of the bacterial core of the oropharyngeal microbiota of smokers and non-smokers when healthy, expressing common cold symptoms or taking antibiotics. Lower left-hand side diagram shows the phylogeny of the pooled data set and the sizes of the circles associated with different taxa indicate their relative abundances. Brown and cyan colours indicate significant differences across pairwise abundances with the colour indicating in which group abundances were greater (with deeper colours indicating higher abundances), while grey represent no significant difference in relative abundance.

To find the relationship between features of microbial communities (abundance tables produced as above) and sources of variation (age, years smoking and cigarettes smoked/week), we have used Generalised Linear Latent Variable Model (1), which extends the basic generalized linear model that regresses the mean abundances μ_{ij} (for i-th sample and j-th microbe) of individual features against environmental covariates x_i as above by incorporating latent variables u_i as $g(\mu_{ij}) = \eta_{ij} = \alpha_i + \beta_{0j} + \boldsymbol{x}_i^T\boldsymbol{\beta}_j + \boldsymbol{u}_i^T\boldsymbol{\theta}_j$, where $\boldsymbol{\beta}_j$ are the features specific coefficients associated with individual covariate (a 95% confidence interval of these whether positive or negative, and not crossing 0 boundary gives directionality with the interpretation that an increase or decrease in that particular covariate causes an increase or decrease in the abundance of the microbe), and $\boldsymbol{\theta}_j$ are the corresponding coefficients associated with latent variable. β_{0j} are microbe-specific intercepts, whilst α_i are optional sample effects which can either be chosen as fixed effects or random effects. To model the distribution of individual features, we have used Negative Binomial distribution. Additionally, the approximation to the log-likelihood is done through Variational Approximation (VA) with final sets of parameters in glvmm() function being family = 'negative.binomial', method="VA", and control.start=list(n.init = 7, jitter.var = 0.1)that seemed to fit well. This, we did for top 100 most abundant OTUs in our datasets. In addition, the factor loadings $\boldsymbol{\theta}_j$ store correlations of microbes with the residual covariance matrix $\boldsymbol{\Sigma} = \boldsymbol{\Gamma}\boldsymbol{\Gamma}^T$ where $\boldsymbol{\Gamma} = [\theta_1 \dots \theta_m]$ for m latent variables. This residual covariance matrix gave co-occurrence relationship between microbes that are not explained by environmental covariates as above.

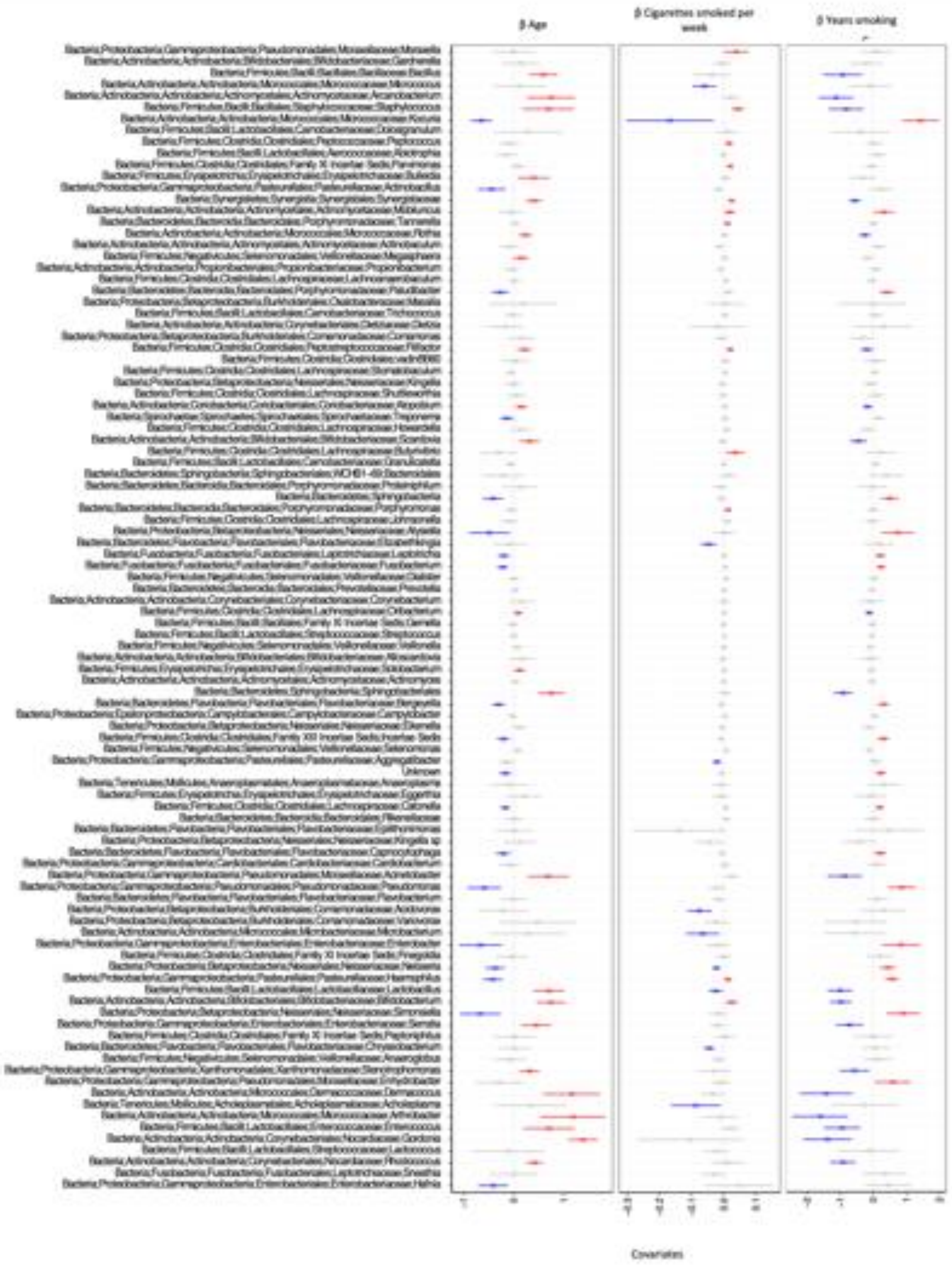

Figure S6. β-coefficients returned from GLLVM procedure for covariates considered in this study by considering top 100 most abundant OTUs incorporating both continuous data (Age, number of cigarettes smoked per week, years smoking) as well as categorical labelling of samples. Those coefficients which are positively associated with the microbial abundance of a particular species are represented in red colour whilst those that are negatively associated are represented with blue colour, respectively. Where the 95% confidence interval of the β-coefficients crosses the 0 boundary, the coefficients are insignificant and are represented by grey color. Those OTUs that cannot be categorized based on taxonomy are collated under “Unknown” category.

Actinobacterium spp. showed significantly negative associations with age as shown elsewhere as shown elsewhere (2). *Micrococcus* spp. showed a positive association the number of cigarettes smoked per week. Higher prevalence of *Micrococcus* spp. in smokers has been shown elsewhere (3, 4). Greater number of cigarettes in particular has a significant positive effect on *Bacillus* spp, *Straphylococcus* spp. and *Arcanobacterium* spp. .We hypothesize that smoking is likely to lead to damage in the mucosal surfaces, allowing these species to colonize.

References

1. Niku J, Hui FK, Taskinen S, Warton DI. 2019. gllvm: Fast analysis of multivariate abundance data with generalized linear latent variable models in r. Methods in Ecology and Evolution 10:2173–2182.

2. Savitt ED, Kent RL. 1991. Distribution of Actinobacillus actinomycetemcomitans and Porphyromonas gingivalis by subject age. Journal of periodontology 62:490–494.

3. Ilankizhai R, Leelavathi L. 2018. Comparison of oral microbiota among smokers and non-smokers-A pilot study. Drug Invention Today 10.

4. Morishita Y. 1983. Mutagenicity of pyrolysates of salt-tolerant bacteria from food and cigarettes. Cancer Letters 18:229–234.
